# Digitally-enhanced dog behavioral testing

**DOI:** 10.1038/s41598-023-48423-8

**Published:** 2023-12-01

**Authors:** Nareed Farhat, Teddy Lazebnik, Joke Monteny, Christel Palmyre Henri Moons, Eline Wydooghe, Dirk van der Linden, Anna Zamansky

**Affiliations:** 1 University of Haifa, Haifa, Israel; 2 Ariel University, Ariel, Israel; 3 University College London, London, UK; 4 VIVES University College, Roeselare, Belgium; 5 Northumbria University, Newcastle upon Tyne, UK

**Keywords:** Animal behaviour, Machine learning

## Abstract

Behavioral traits in dogs are assessed for a wide range of purposes such as determining selection for breeding, chance of being adopted or prediction of working aptitude. Most methods for assessing behavioral traits are questionnaire or observation-based, requiring significant amounts of time, effort and expertise. In addition, these methods might be also susceptible to subjectivity and bias, negatively impacting their reliability. In this study, we proposed an automated computational approach that may provide a more objective, robust and resource-efficient alternative to current solutions. Using part of a ‘Stranger Test’ protocol, we tested n = 53 dogs for their response to the presence and neutral actions of a stranger. Dog coping styles were scored by three dog behavior experts. Moreover, data were collected from their owners/trainers using the Canine Behavioral Assessment and Research Questionnaire (C-BARQ). An unsupervised clustering of the dogs’ trajectories revealed two main clusters showing a significant difference in the stranger-directed fear C-BARQ category, as well as a good separation between (sufficiently) relaxed dogs and dogs with excessive behaviors towards strangers based on expert scoring. Based on the clustering, we obtained a machine learning classifier for expert scoring of coping styles towards strangers, which reached an accuracy of 78%. We also obtained a regression model predicting C-BARQ scores with varying performance, the best being Owner-Directed Aggression (with a mean average error of 0.108) and Excitability (with a mean square error of 0.032). This case study demonstrates a novel paradigm of ‘machine-based’ dog behavioral assessment, highlighting the value and great promise of AI in this context.

## Introduction

Behavioral traits in animals are consistent patterns of behaviors exhibited across similar situations^1–4^. They are driven by personality^5^, which is a complex combination of genetic, cognitive, and environmental factors^6^. The assessment of personality traits in dogs is gaining increasing attention due to its many practical applications in applied behavior^7–10^. Some examples of such applications include determining the suitability of dogs for working roles^11–13^, identifying problematic behaviors^14^, and adoption-related issues for shelter dogs^4,15,16^.

Measuring behavioral traits of dogs has been an enigmatic challenge in scientific literature for decades. Two of the most common methods are behavioral testing and questionnaires. The former refers to experimental behavioral tests (e.g., observations of the dog’s behavior in a controlled novel situation, such as the Strange Situation Test^17^). Such tests can be rated, scored and assessed using standard ethological methods of behavioral observation^18,19^. Brady et al.^20^ provide a systematic review of the reliability and validity of behavioral tests that assess behavioral characteristics important in working dogs. Jones and Gosling^21^ provide another comprehensive review of past research on canine temperament and personality traits. In a complementary manner, Bray et al.^12^ reviewed 33 empirical studies assessing the behavior of working dogs. Tests for detection dogs have also been addressed^22–24^. The latter method refers to questionnaires completed by the owner or handler. Examples include the Monash Canine Personality Questionnaire^25^, the Dog Personality Questionnaire^26^ and many more. One of the most well-known questionnaires, used in many contexts, is the Canine Behavioral Assessment and Research Questionnaire (C-BARQ). Originally developed in English^27,28^, it has been validated in a number of languages.

Although questionnaires are more time and resource efficient, and can better represent long-term trends in behavior compared to behavioral testing, they have serious limitations: they are susceptible to subjectivity and misinterpretation, and can be biased by the bond with the animal being assessed.

In the context of owner-observed assessment of stress, Mariti et al.^29^ have argued that many owners would benefit from more educational efforts to improve their ability to interpret the behavior of their dogs. Kerswell et al.^30^ also showed that owners often overlook some subtle cues dogs exhibit in the initial phases of emotional arousal. Even seemingly clear physical observations, such as obesity in dogs, have been shown to lead to frequent disagreements between owners and veterinarians^31^. Moreover, in the case of working or shelter dogs, individuals with sufficient knowledge of the dog are not always available to complete questionnaires^20^.

Rayment et al.^32^ criticize the lack of proper assessment of the validity and reliability of many test tools. These include psychometric instruments that rely on an unambiguous shared understanding of terminology, which is difficult to achieve in a population with different levels of knowledge about animal behavior. Psychological factors of the human observers influence their evaluation of dogs too^33^, which further complicates the use of psychometric data from a wide variety of participants as a homogenous dataset of observations.

The goal of this exploratory study was to investigate a novel idea of a *digital enhancement* for behavioral testing, which in time may be integrated into relevant interspecies information systems^34^ to understand animal behavior. In other words, we study how ‘the machine’, or machine learning algorithms, may help human experts in behavioral testing. Using as a case study a simple behavioral testing protocol of coping with the presence of a stranger, currently implemented to improve breeding of working dogs in Belgium, we asked the following questions:

- Can the machine identify different ‘behavioral profiles’ in an objective, ‘human-free’ way, and how do these profiles relate to the scoring of human experts in this test?
- Can the machine predict scoring of human experts in this test?
- Can the machine predict C-BARQ categories of the participating dogs?

## Methods

### Ethical statement

All experiments were performed in accordance with relevant guidelines and regulations. The experimental procedures and protocols were reviewed by the Ethical Committees of KU Leuven and University of Haifa, in both ethical approval was waived. Informed consent was obtained from all subjects and their legal guardians.

**Figure 1:**
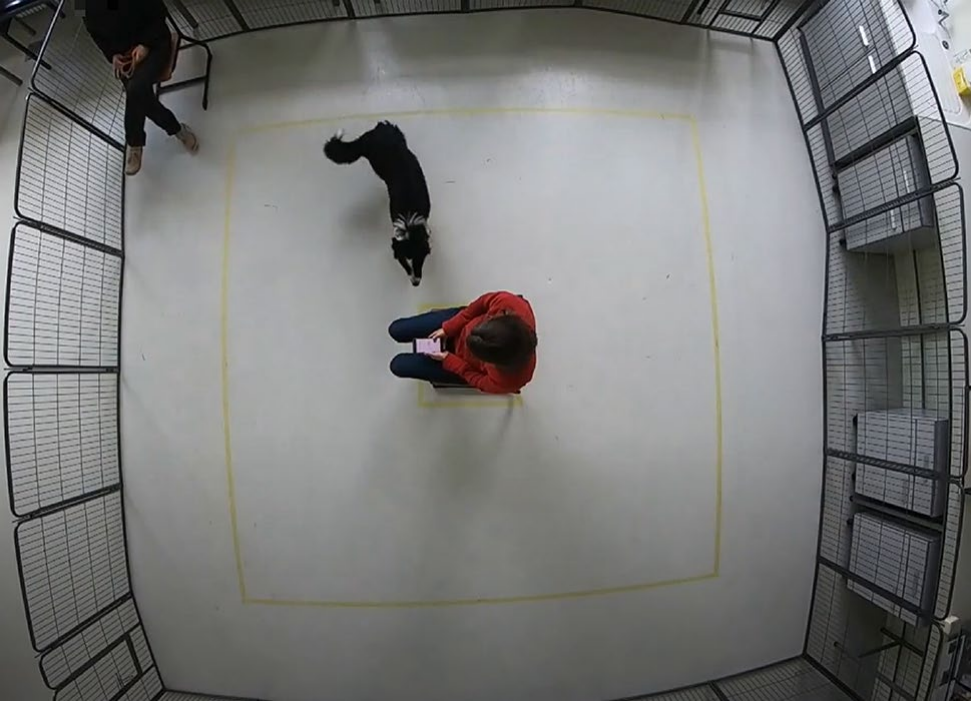
A frame collected of the testing arena used to record dogs’ behavior during the ’Stranger test’. The arena was fenced and recorded from above using a camera. The test person (stranger) is sitting in the middle, and the familiar person (owner) is sitting in the corner.

### Testing arena

The test was conducted indoors, in a room free from other distractions such as other animals or people, with exception of the test person (TP), the assistant, and the familiar person i.e., the owner or trainer (FP). The testing room, of size 8 m $\times$ 6 m contained a testing arena surrounded by a fence made of metal wires of height of 0.8 m, and size of 4.7 $\times$ 4.7 m, with a gate entrance towards the area where the assistant was located. In the middle of the test arena, a square of 60 $\times$ 60 cm was drawn with tape for positioning the chair of the test person at a fixed location. The test person (TP) faced the gate entrance. In the left corner (frontal view), there was a chair for the familiar person (FP), positioned parallel to the front fence. A second square of size 3 $\times$ 3 m, centered around the TP chair, was marked with tape on the floor. These lines indicated the track to be followed when the owner or test person walked in the test arena. An adjacent, separate room was available where the dog and the owner were received and could wait out of sight of the testing arena. The TP could enter the testing arena without being seen by the dog and owner, so that TP was novel for the dog until the start of the actual test.

**Figure 2:**
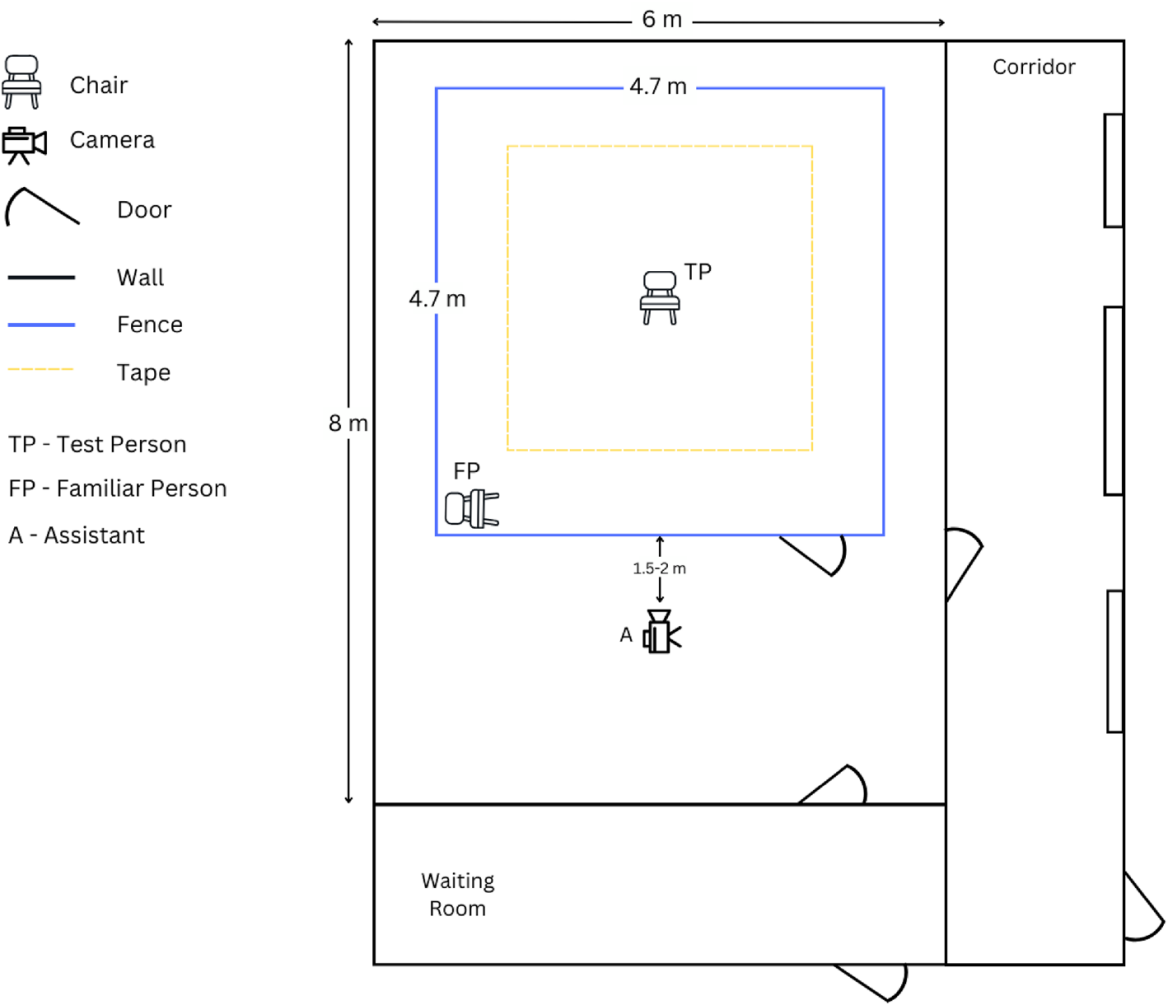
A schematic drawing of the testing room and arena with sizes and locations of test person, familiar person, assistant, entrances and exists.

Figure 1 presents a view from above on the testing arena; Fig. 2 shows the experimental setting in further details.

**Figure 3:**
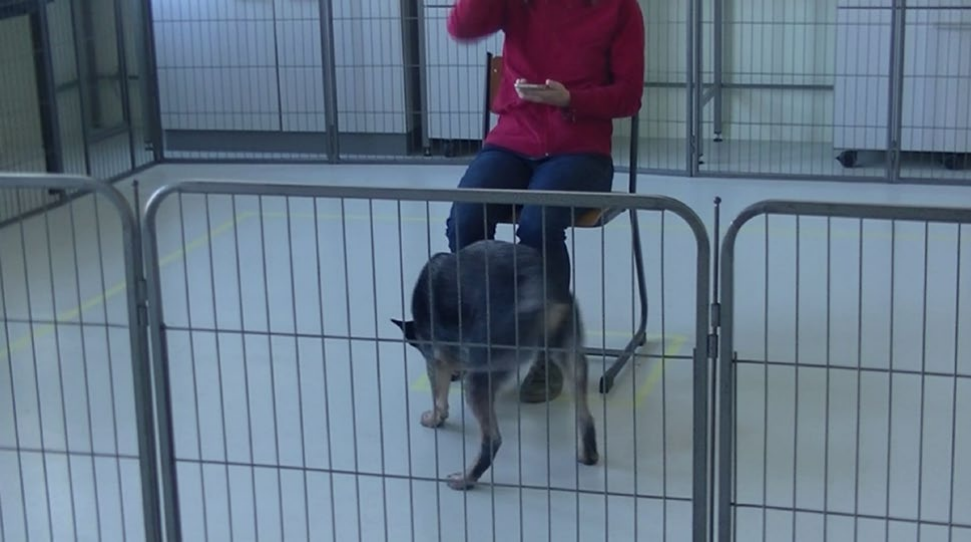
A frame of the testing arena captured from the side camera held by the assistant. The assistant recorded the dog closely for the entire testing phase.

Two video cameras were used to record the activity and the behavior of the dog during the test, a top view and a side view camera. As top view, a GoPro Hero 7 video camera was mounted in the middle of the test arena at a height of approximately 3 m, so that the entire test arena was covered—see Fig. 1. A side-view camera (JVC Quad proof, full HD) was held and operated by the assistant, recording more nuanced behaviors used for expert scoring; Fig. 3 shows the view from the side camera; Fig. 2 shows the locations of the TP, FP and assistant.

### Test procedure

The protocol used below was a part of a more elaborate testing protocol developed by one of the authors (JM), starting with an exploration phase, to reduce the novelty of the environment for the dog, and followed by 16 test phases. The purpose of the latter was to assess the reactions of dogs to the presence of an unfamiliar person (during inactivity or during neutral and benign actions), both in the presence and absence of a familiar person (i.e., the owner or a regular handler/trainer). This study includes only the first test phase of the full protocol, from now on referred to as the “testing phase”. This phase consisted of inactivity and neutral actions, only in the presence of a familiar person.

Prior to the testing phase, the familiar person was instructed about the test and asked not to interact with the dog. When starting the testing phase, the assistant called in the familiar person and the dog in the test arena, where the test person was seated on the chair in the middle, feet in parallel and firmly planted. The test person held a smartphone as a timer. The familiar person closed the gate of the test arena, unleashed the dog, walked directly to the chair and sat down. After the familiar person sat down, the test person performed three actions: a short, clear cough (at 10 s), a hand running through the hair for 3 s (at 20 s), and crossing the right leg over the left (at 30 s). These are neutral actions that can be expected from any human being and that all dogs will encounter when they are around people. An example trial can be found here (https://drive.google.com/file/d/1VpaxKePw2ICY2SMGPwK_T4Td2zGyAOzc/view?usp=sharing). Except when running her hand through her hair or when a dog jumps up, the test person held the smartphone in both hands, resting on her lap. The test person did not look at the dog or perform any actions towards it. If a dog jumped up excitedly, the test person protected her face/head with her hands/arms as needed. During this phase, the whole testing arena was filmed by the camera in top view and the behavior of the dog was filmed in side view by the assistant. Subsequently, both videos were used further in this study for dog scoring by the experts and the computational approach.

The unfamiliar person, i.e., the test person, was always the same adult female (JM). The assistant was also always an adult female, but not always the same person. As most of the testing took place at the time of COVID pandemic, the testing person, familiar person and assistant were wearing masks during the test, except for five dogs when masks were no longer obligatory.

#### Study subjects

A total of n = 53 dogs were tested in the study. Their owners were recruited through social media in Belgium. The inclusion criteria for the dogs were:

- Age: between 11 and 24 months old.
- Height: between 30 and 65 centimeters.
- Up-to-date vaccinations and no history of health problems.
- Accompanied by a familiar person.
- Belonging to the modern dog breeds.

Demographic data on the participants is provided in Appendix 1.

#### Dog scoring

Dogs were scored using the scoring method previously developed by JM for the Belgian assistance dog breeding organization Purpose Dogs vzw (https://purpose-dogs.be/) to improve breeding outcomes. The method is based on an adaptation of the concept of coping with potential threats via freeze/flight versus fight^35^. The original scoring method used an eleven-point scale ranging from − 5 to + 5. However, for the purposes of our study, the scale was simplified to a five-point scale ranging from − 2 to + 2. The positive/negative scores aimed to differentiate between two main tendencies of dogs when reacting to a stressor (in this context, an unfamiliar person; reactions to the assistant and the FP were ignored): dogs that tended to ‘react towards the stressor’ (e.g., get very close to the test person, jump up, chew, show offensive aggression) received numerically positive scores, and dogs that tended to ‘react away from the stressor’ (e.g., keep at a distance, avoid, show defensive aggression) received numerically negative scores. A larger absolute value for a score indicated a stronger response by the dog (either reacting towards (‘+’) or away from (‘−’) the stressor). Thus negative scores (− 2 for fleeing away from the TP, or extremely frozen, and − 1 for keeping a distance and avoiding the TP) referred to reacting away from the stressor, while positive scores (+ 2 for biting and jumping on TP, + 1 for approaching and continuously interacting with TP) referred to reacting towards the stressor. The score 0 (neutral) indicated mostly neutral and stable coping with the stressor, slowly approaching and sniffing the TP, and then moving on exploring further. The analysis for the purpose of this study was further simplified by grouping the negative (− 2 and − 1) and positive (+ 2 and + 1) scores, respectively, resulting in three groups: “+” , “0”, and “−”.

The testing phase was evaluated by three dog behavior experts (JM, CPHM, EW); the dog received one overall score for the entire phase. To measure reliability of scoring, multi-rater (Fleiss) kappa was used. In case of disagreement among expert scores, the final score was aggregated using majority voting. For example, if two experts scored “+” and one scored “0”, the dog would receive a score “+”. Since only three dogs had negative scores, the negative category was excluded from our analysis due to its small number of samples. Our final dataset included 50 samples, of which 32 samples with a zero/neutral score (26 full agreement by all coders, 6 by majority) and 18 samples with a positive score (12 full agreement by all coders, 6 by majority).

#### C-BARQ questionnaire

The Canine Behavioral Assessment and Research Questionnaire (C-BARQ) is a questionnaire for owners/handlers to rate the behavior of their dog in various contexts and related to different behavior aspects, such as stranger and owner directed aggression, social and non-social fear, separation related behavior. An instrument originally developed in English^27,28^, it has been translated to and validated in multiple languages, including Dutch^36^.

In the context of our study, we used the following eight CBARQ categories identified in Hsu et al.^27^: Stranger directed aggression (SDA), Owner directed aggression (ODA), Stranger directed fear (SDF), Nonsocial fear (NSF), Separation related behavior (SRB), Attachment seeking behavior (ASB), Excitability (EXC), and Pain sensitivity (PS).

The dog owners were asked to complete a Dutch version of the C-BARQ questionnaire.

### Computational approach

**Figure 4:**
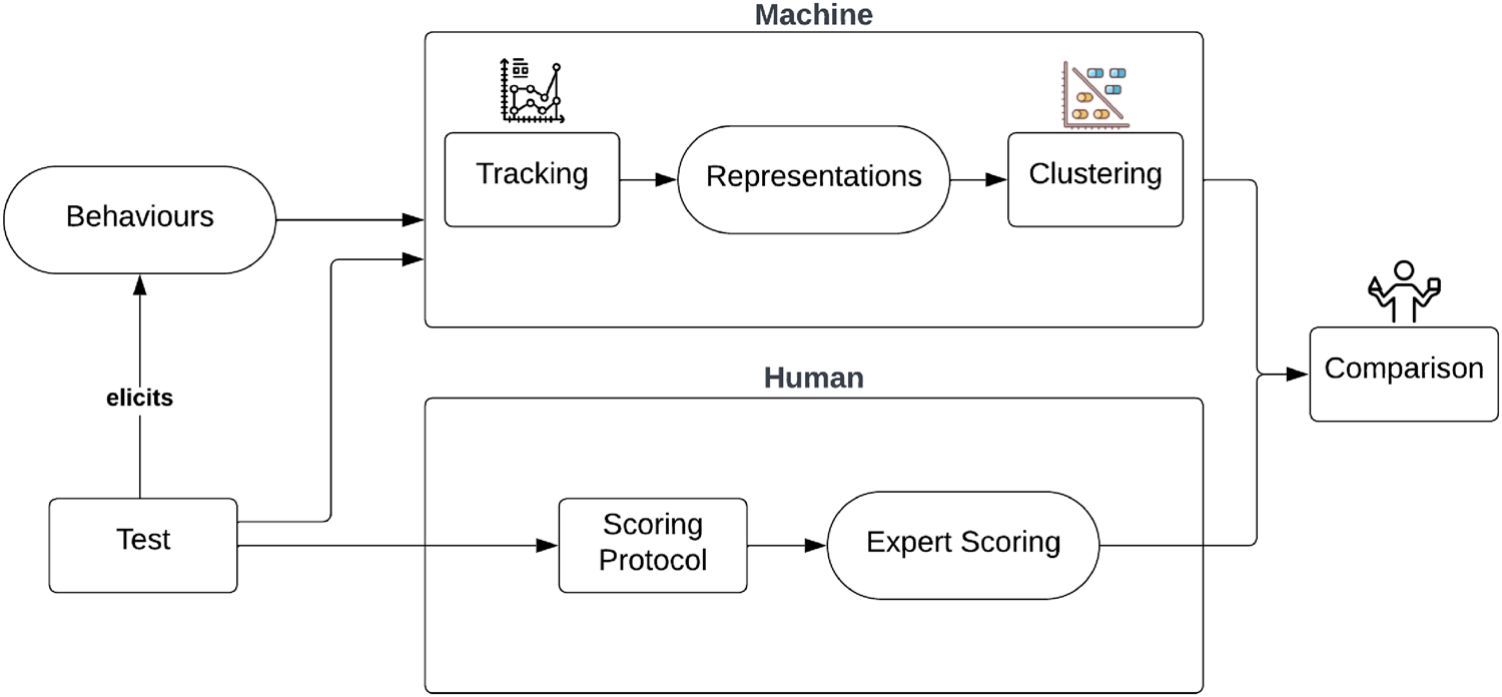
A conceptual framework for digitally enhanced dog behavioral assessment.

The purpose of any behavioral test is, eventually, to observe behaviors in response to various stimuli in a controlled and standardized environment. Based on a specific testing protocol, a scoring method is usually developed and evaluated for use by human experts. The practical aim of such scoring is to classify the elicited behaviors into categories (e.g. corresponding to specific behavioral traits or profiles) that can eventually be used for decision support. With the machine entering the scene, we have an alternative, mathematical and *completely human-free* way of “scoring” behaviors, or dividing them into categories. Since this test focuses on human-directed behavior, we assume that the participants’ trajectories contain meaningful behavioral information about their reaction to the stranger. Therefore, we automatically extract and cluster the dogs’ trajectories, investigating the relationship of the emerging clusters to experts’ scoring, and compare how well they align. This process is demonstrated in Fig. 4, which provides an overview of this conceptual framework for digital enhancement of dog behavioral assessment.

Further details on the tracking method, the clustering method, the machine learning models for prediction of the above and statistical analysis to compare clustering with C-BARQ are given below

#### Tracking method

The BLYZER system is a self-developed platform that aims to provide a flexible automated behavior analysis which has been applied in several studies for analyzing dog behavior^37–40^. A similar approach was implemented on a smaller portion of the dataset used in this study in^41^, however in contrast to our approach here, features chosen manually were used for clustering.

**Figure 5:**
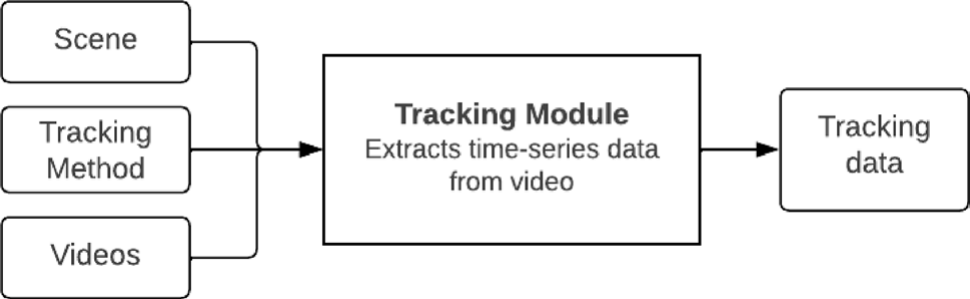
BLYZER tracking module architecture.

**Figure 6:**
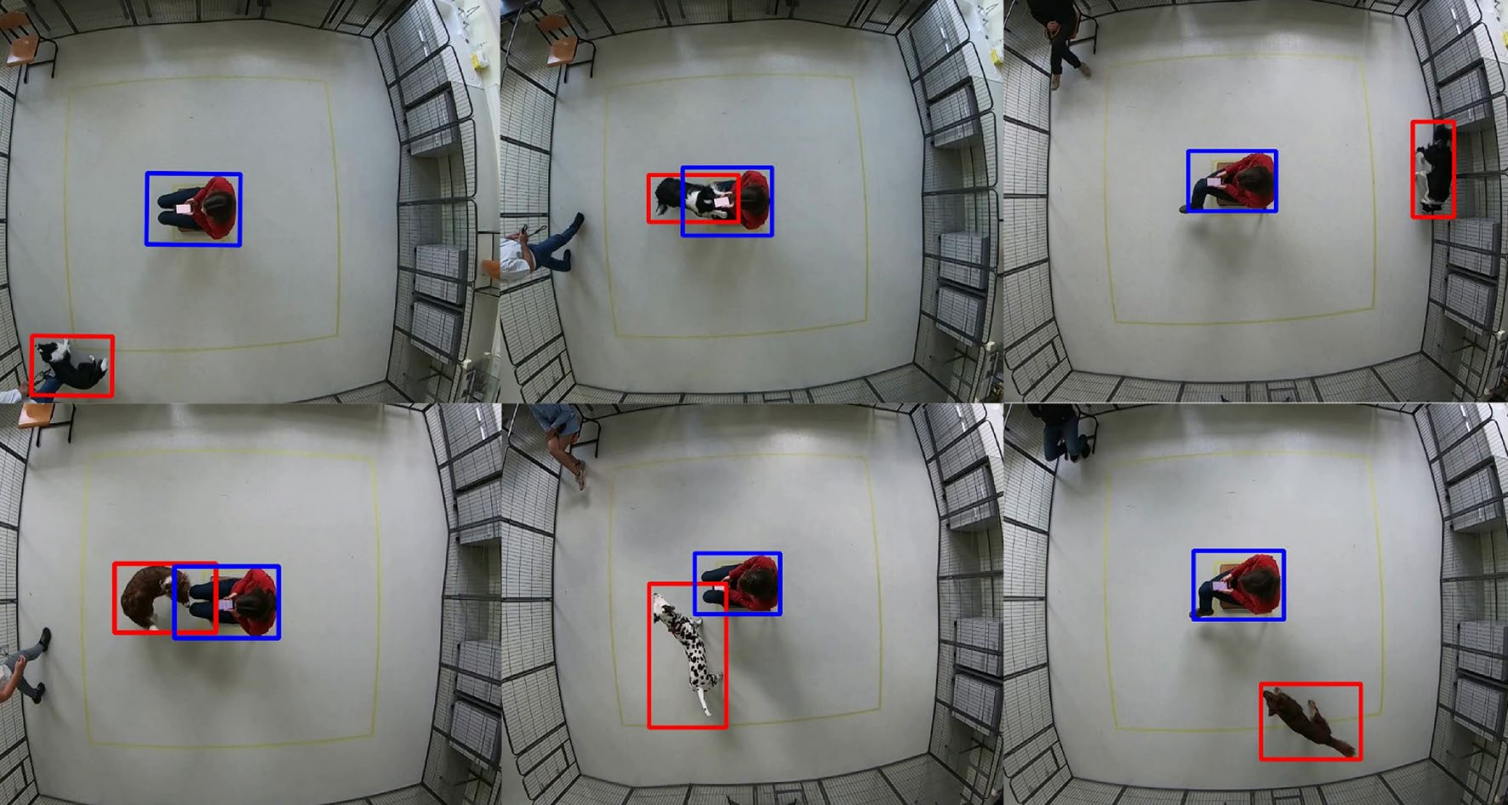
Example of frames extracted from the test recording, showing the participating dog and test person being detected by Blyzer.

**Figure 7:**
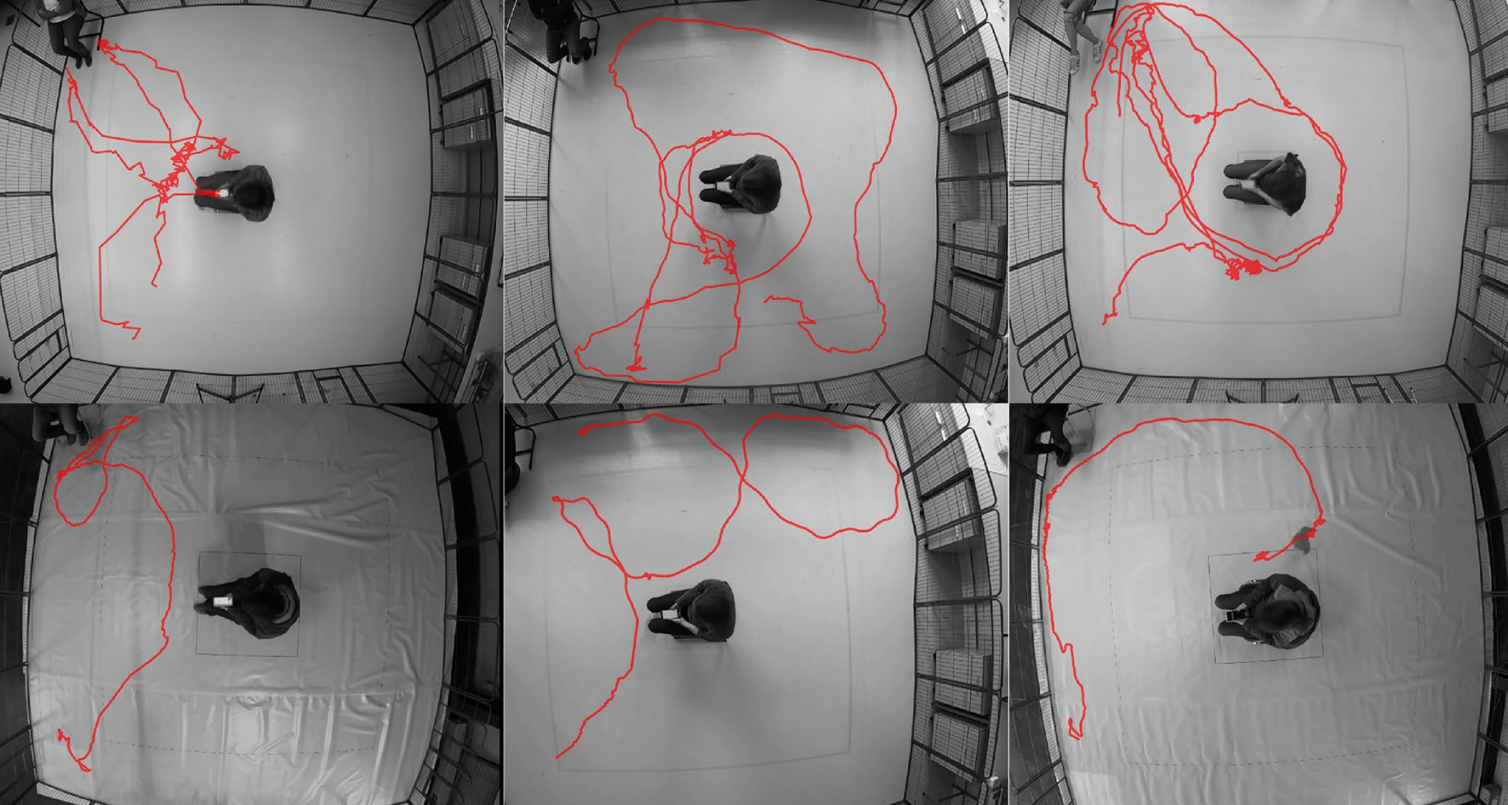
Examples of participating dogs’ trajectories extracted with BLYZER and printed on an extracted frame from the recorded test; top: scoring ‘+’, bottom: scoring 0.

BLYZER’s input is video footage of a dog freely moving in a room and possibly interacting with objects, humans or other animals, while its output is time series (representing the dog’s trajectory) in a json file with the detected locations of the objects in each frame. Figure 5 shows the pipeline. Both the tracking method (the models used for detection) and the scene (amount of moving and fixed objects) can be adapted to the specific study. In our setting, e.g., the scene consists of one moving object (dog) and one static object (TP). The tracking method was chosen to be a neural network based on the Faster R-CNN architecture^42^ pre-trained on the COCO 2017 dataset^43^, which we retrained on additional 106,768 images of two objects: a person and a dog. The images were collected from (1) Open image dataset V6^44^ (2) Pascalvoc dataset^45^ (3) COCO dataset^43^ (4) Images from previous studies^39,40^. Figure 6 shows example frames from our dataset with dog and test person object detection. And Fig. 7 presents examples of dogs’ trajectories extracted with BLYZER.

*Quality of detection.* To ensure sufficient tracking, only videos with a percentage of frames where dog and person are correctly detected of least 80% of the frames, leading to the exclusion of three videos (all three scored with a zero/neutral score). For the remaining 47 videos, we applied post-processing operations available in BLYZER to remove noise and enhance detection quality using smoothing and extrapolation techniques for the dog and test person detection, reaching almost perfect (above 95%) detection.

### Clustering method

**Figure 8:**
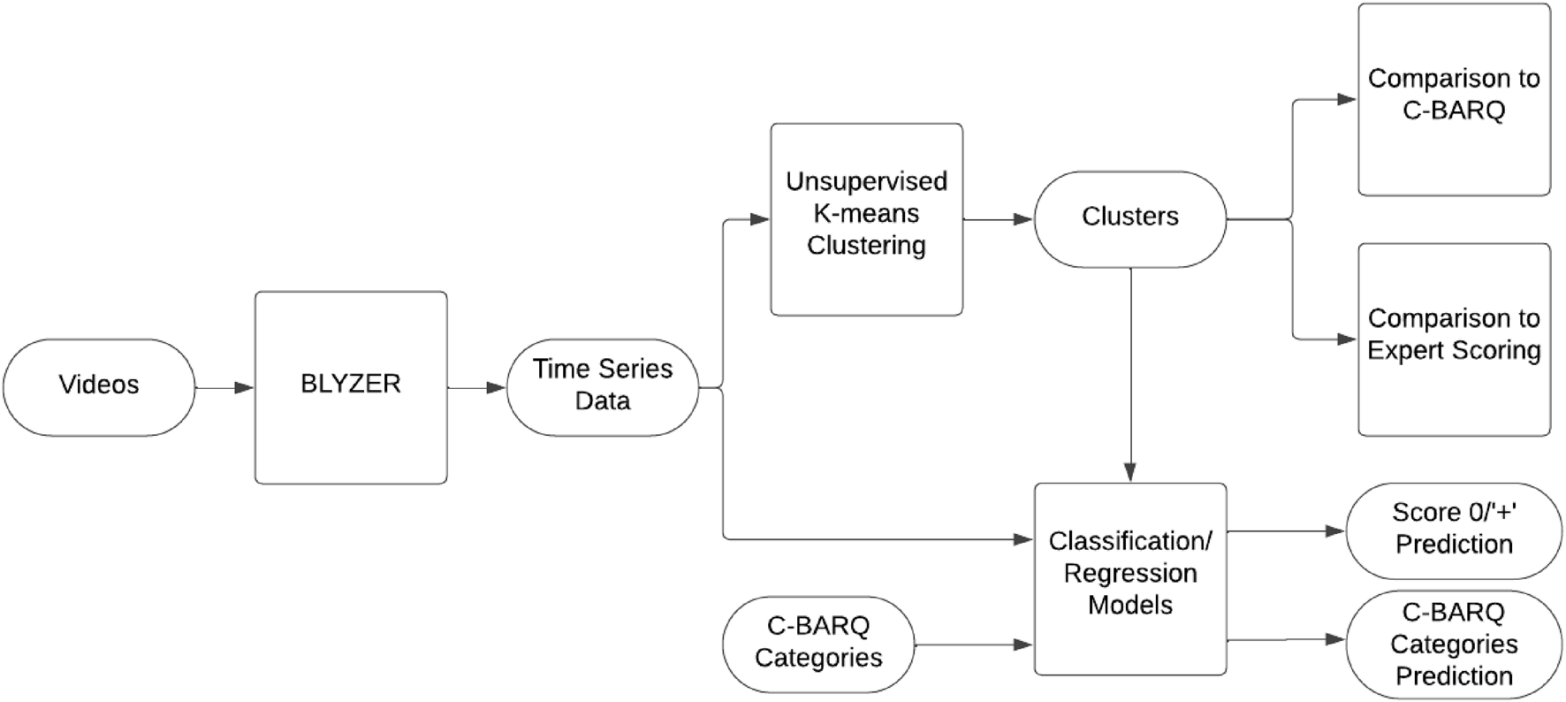
Data analysis pipeline.

The videos from the trials are initially analyzed by the BLYZER tool which produces for each frame the center of mass of the dog and person in the frame (if detected). To assure a smooth motion capture while standardizing between trials, we set 24 frames per second (FPS) rate across all videos. For frames that the BLYZER tool was not able to detect either the dog or the person (or both), it linearly extrapolates their positions to fulfill the gap. In addition, since not all videos were of identical duration, we used the duration of the shortest video as standard duration. As such, each trial ($s \in \mathbb {R}^{2m}$) is defined by a time series with a fixed duration between samples constructed by two vectors, one for the dog’s position $(d \in \mathbb {R}^m)$ and the other for the person’s position $(p \in \mathbb {R}^m)$. As a result, we obtain a dataset, $D \in \mathbb {R}^{n \times m}$. This is the times series data depicted in Figure 8, which presents the whole data analysis pipeline.

For clustering trajectories, we used the time-series K-mean clustering algorithm^46^ with the elbow-point method^47^ to find the optimal number of clusters ($k$). Nonetheless, as the raw center of mass is not quite an informative space, we decided to first transform the data into a “movement” space. To this end, we trained a small-size one-dimensional convolutional neural network (CNN) based AutoEncoder model^48^ with the following architecture for the encoder: Convolution with a window size of 3, dropout with $p = 0.1$, max-pooling with a window size of $2$. Clearly, the decoder’s architecture is opposite to the encoder’s one. We used a mean absolute error as the metric for the optimization process and the ADAM optimizer^49^. The model’s hyperparameters are found using a grid-search^50^. Using the encoder part of the model that was used after training the AutoEncoder, we computed the “movement” space of each sample for the clustering. Once the clustering is obtained, the clusters were evaluated against expert scoring metrics. T-SNE method with a normalization between 0 and 1 was used to visualize the clusters.

### Statistical analysis

Mann Whitney U test was performed to compare the means of the C-BARQ scores between the obtained clusters for each of the C-BARQ categories (1, Stranger directed aggression (SDA); 2, Owner directed aggression (ODA); 3, Stranger directed fear (SDF); 4, Nonsocial fear (NSF); 5, Separation related behavior (SRB); 6, Attachment seeking behavior (ASB); 7, Excitability (EXC); and 8; Pain sensitivity (PS)).

### Classification and regression machine learning models

The clustering was further used to obtain classification and regression models for predicting scoring (0/‘+’) and C-BARQ categories, respectively. We use the Tree-Based Pipeline Optimization Tool (TPOT), the genetic algorithm-based automatic machine learning library^51^. TPOT produces a full machine learning (ML) pipeline, including feature selection engineering, model selection, model ensemble, and hyperparameter tuning; and shown to produce impressive results in a wide range of applications^52–54^. Hence, for every configuration of source and target variables investigated, we used TPOT, allowing it to test up to $10,000$ ML pipelines. We choose $10,000$ to balance the ability of TPOT to converge into an optimal (or at least close to optimal) ML pipeline and the computational burden associated with this task.

The obtained classification model performance for expert scoring was evaluated using commonly used metrics of accuracy, precision, recall, and $F_1$ score. The obtained regression model performance for C-BARQ categories was evaluated using Mean Absolute Error^55^ (MAE), Mean Squared Error^55^ (MSE), and R-squared^56^ ($R^2$).

## Results

### Inter-rater reliability of expert scoring

Multi-rater (Fleiss) kappa on the scores (n = 53) collapsed into three classes (negative‘−’, neutral 0, and positive ‘+’) reached a percentage of agreement of 85%; Fleiss free-marginal k = 0.77 indicating good strength of inter-rater reliability.

### Clusters vs. expert scoring

Using the elbow method, two clusters emerged of sizes 26 and 20 respectively. One sample was excluded due to being an outlier. As shown in Table 1, there is a quite good separation between zero/neutral scores and positive/excessive scores: the first cluster had the majority of participants (n = 21) scoring 0, while only 5 scored ‘+’. The second, the majority (n = 13) scored ‘+’ while 7 scored 0.

**Figure 9:**
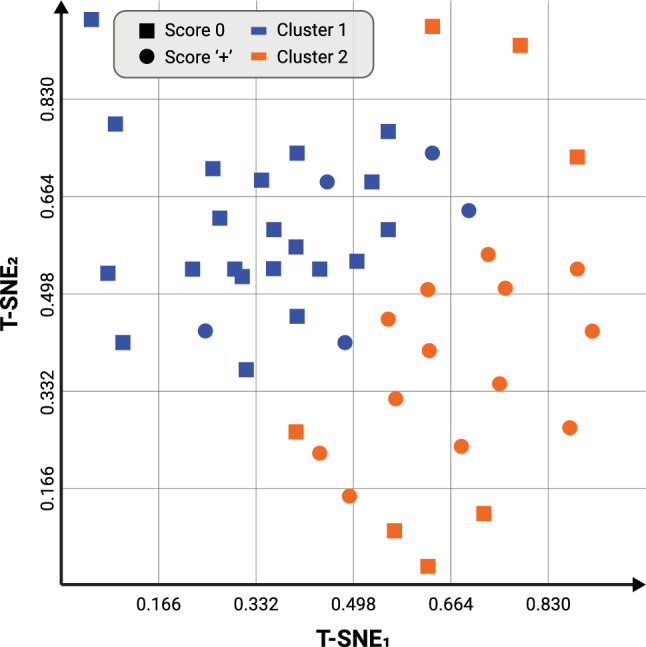
A visualization of the clusters vs. domain expert agreement for $n = 46$ dogs. The axis are obtained using the T-SNE method and normalization between 0 and 1.

To visually demonstrate the relationship between the domain experts’ scoring and the computationally obtained clusters, Fig. 9 provides a visualization of the clusters and domain expert agreement for n = 46 dogs. The axis of the figures are obtained using the T-SNE method and normalization between 0 and 1. The shape represents the expert scoring (circles for score 0, squares for score ‘+’) while the color represents the resulting cluster (blue for cluster 1, orange for cluster 2). The blue circles and orange squares represent the dogs that were clustered ‘incorrectly’.

**Table 1:** Cluster description in correlation with expert scoring.

Expert scoring vs. clusters	Cluster 1	Cluster 2	Total
Score 0	21	7	28
Score ‘+’	5	13	18
Total	26	20	46

### Clusters vs. C-BARQ

There was a significant difference between the two clusters with respect to Stranger-Directed Fear (SDF) with a median of 0.00 for cluster 1 and 0.42 for cluster 2 (Mann Whitney U = 120.5, z = − 2.56, p = 0.01). No other categories in the C-BARQ showed a meaningful variation between the clusters.

### Automating expert score classification

Table 2 presents the performance of the expert score classification model, reaching accuracy of above 78%.

**Table 2:** Evaluation metrics of the expert scoring classification model.

	Precision	Recall	F1	Accuracy
Score classifier	0.771	0.782	0.775	0.787

### C-BARQ categories regression model

Our findings revealed varying levels of error across the eight C-BARQ categories, the metrics are summarized in Table 3. Owner directed aggression (ODA) and Excitability (EXC) exhibited the lowest errors in terms of MAE and MSE respectively. The $R^2$ values provided insights into the proportion of variance explained by each category. Notably, EXC demonstrated the highest $R^2$ value, indicating a strong fit between the EXC category and the time-series data. SDA and PS exhibited moderate $R^2$ values, signifying a reasonable level of explanatory power. These outcomes illuminate on the predictive performance of the model and highlight the varying impacts of the C-BARQ categories on the outcome.

**Table 3:** Regression model metrics per C-BARQ category.

	MAE	MSE	$R^2$
Owner directed aggression (ODA)	**0.108**	0.046	0.176
Excitability (EXC)	0.122	**0.032**	**0.886**
Separation related behavior (SRB)	0.257	0.144	0.073
Stranger directed aggression (SDA)	0.275	0.129	0.470
Pain sensitivity (PS)	0.435	0.319	0.429
Non social fear (NSF)	0.438	0.334	0.043
Attachment seeking behavior (ASB)	0.441	0.287	0.142
Stranger directed fear (SDF)	0.510	0.430	0.032

Maximal values are in [bold].

## Discussion

This study is another contribution to the growing field of computer-aided solutions for “soft” questions using data-driven based methods^57–61^. To the best of our knowledge, this study is the first to provide a machine-learning model for objectively scoring a strictly controlled dog behavioral test.

In this study we used a Stranger Test routinely performed in a working dog organization, as a case study, to ask the following questions:

- Can the machine identify different ‘behavioral profiles’ in an objective, ‘human-free’ way, and how do these profiles relate to the scoring of human experts in this test?
- Can the machine predict scoring of human experts in this test?
- Can the machine predict C-BARQ categories of the participating dogs?

Our results indicate positive answers to all of the above questions. Answering the first question, using unsupervised clustering, two clusters emerged, with a good separation between the group with score 0 and the group with score ‘+’. Answering the second question, we presented a classification model for predicting human scoring reaching 78% accuracy. Answering the third question, we presented a regression model which is able to predict C-BARQ category scores with varying performance, the best being Owner-Directed Aggression (with a mean average error of 0.108) and Excitability (with a mean square error of 0.032).

It is important to stress that the computational approach to the assessment of dog behavioral testing proposed here is ‘human-free’. The agenda for a ‘human-free’ computational analysis of animal behavior was introduced in Forkosh^62^. The author argued that despite the fact that automated tracking of animal movement is well-developed, the interpretation of animal behaviors remains human-dependent and thus inherently anthropomorphic and susceptible to bias. Indeed, in previous works applying computational approaches in the context of dog behavior^37,40,63,64^, features used for machine learning are explicitly selected by human experts.

By using such “human-free” clustering, two clusters emerged, roughly dividing the participants into a cluster of ‘neutrally reacting’ dogs with the majority scoring 0, and a cluster with a majority of ‘excessively reacting’ dogs scoring ‘+’. Interestingly, these clusters showed a significant difference in the Stranger-Directed Fear C-BARQ category. However, a regression model for predicting this category did not have a very good performance, with the best performance being the Owner-Directed Aggression category and Excitability. The latter could be related to the excessive behaviors typical of the ‘+’ scoring that matched the response of dogs as measured by the C-BARQ “displaying strong reactions to potentially exciting or arousing events”^65^. Further research is needed to establish clearer relationships.

The testing protocol used in our study refers to one specific aspect (towards/neutral/away from stressor) of stranger-directed behaviors. This protocol is used in a working dogs organization for breeding outcome improvement and has been previously studied in the context of automation of tracking^63^, also exploring some preliminary ideas of clustering (unlike the ‘human-free’ approach presented here). An in-depth exploration and scientific validation of this test is beyond the scope of the current study, we chose to use just one phase of this protocol due to its simplicity for automating tracking.

A note on the use of C-BARQ questionnaires in this study is in order. Although C-BARQ is a validated and commonly used questionnaire, not only is it subjective due to it being completed by owners or other familiar persons, but also it does not refer to the particular testing situation created in this study, but more generally to the dog. Despite this, the relationship between clusters and the C-BARQ categories of Owner-Directed Aggression and Excitability may indicate that the particular testing protocol used is indeed useful in separating excitable and/or aggressive dogs. How sensitive the results are to variations in the protocol is also a question we plan to explore by repeating the same analysis for other phases of this protocol which involve movement of the TP around the arena.

This study is exploratory, and one of its main limitations is its relatively small number of participants, in which we had an insufficient number of participants with negative scores (reacting ‘away from TP’), thus excluding them from the study. Having a larger representative sample of such dogs is expected to affect the results and should be explored in the future.

In future research, we plan to address other phases of this protocol, which were excluded from the current study. We will also use the side view camera footage that was obtained for manually coding and correlated nuanced behaviors (such as gazing at a stranger, lip licking, etc.) to enhance the analysis performed in this study. Finally, we will look into replacing and/or enhancing video analysis with wearable sensor data, which may be a more feasible approach to be used in the field for behavioral assessment.

Our approach in this study was validating the emerging clusters using expert scoring as a golden standard. However, this approach could be reversed in future studies, using mathematical, objective clustering as a ‘ground truth’ for testing various scoring schemes for behavioral testing protocols. For now, we treat the machine as enhancing human capabilities, however a day may come when this situation will be reversed, with the machine being the more objective and reliable way of analyzing behavioral testing data. It is our hope that this preliminary study will stimulate discussions on the value and great promise of AI in the context of dog behavioral testing.

To summarize, in this study we proposed a machine learning algorithm for the prediction of expert scoring of a behavioral ‘stranger test’ for dogs. The algorithm reached above 78% accuracy, demonstrating the potential value digital enhancement may have in behavioral testing of dogs. We plan to extend this approach to a larger dataset, to consider other protocols, and study the test-retest reliability of the approach.

### Supplementary Information

Supplementary Information.

## Data Availability

The datasets used during the current study are available from the corresponding author upon request.
