# Supplementary material for: Digitally-enhanced dog behavioral testing
Source: Sci Rep. 2023 Dec 1;13:21252. doi: 10.1038/s41598-023-48423-8 (PMC10692085; doi:10.1038/s41598-023-48423-8)
Supplement: Supplementary file 1 — Supplementary Information. [file 41598_2023_48423_MOESM1_ESM.pdf]

Appendix 1

Participants breed, gender and age in years at time of test.

Dog ID	Race or mixed breed	Gender	Dog age (years)
D001	Cattle dog	M	2.0
D002	Kooiker (Dutch Spaniel)	F	1.8
D003	Border Collie	M	1.8
D004	Border Collie	F	1.7
D005	Border Collie	F	1.7
D006	Dalmatian	M	1.5
D007	English Bulldog	F	1.4
D008	Old German Herding Dog	F	1.4
D009	Pug	F	1.4
D010	Border Collie	M	1.3
D011	Border Collie	F	1.3
D012	Border Collie	M	1.3
D036	Labrador Retriever (Blond)	F	1.8
D037	Labrador Retriever	F	1.8
D038	Golden Retriever	M	1.5
D039	Labrador Retriever	M	1.5
D040	Golden Retriever	M	1.6
D041	English Cocker Spaniel	M	0.9
D052	Labrador Retriever	M	1.4
D054	Labrador Retriever	F	1.4
D055	Labrador Retriever	F	1.4
D056	Labrador Retriever	F	1.4
D058	Labrador Retriever	F	1.4
D059	Labrador Retriever	F	1.1
D061	Labrador Retriever	F	1.0
D062	Labrador Retriever	M	1.0
D064	Labrador Retriever	F	1.0
D065	Labrador Retriever	F	0.9
D067	Labrador Retriever	F	1.1
D074	Dachshund	F	0.9
D081	Jack Russell	M	1.0
D088	Border Collie	M	1.7

D099	Mixed Breed	F	1.4
D100	Shetland Sheepdog	F	1.0
D103	Bernese Mountain Dog	F	1.8
D104	White Swiss Shepherd Dog	M	1.5
D105	Golden Retriever	F	1.3
D106	Toy Poodle	F	1.8
D107	American Staffordshire Terrier	F	1.5
D114	English Cocker Spaniel	F	1.0
D115	Australian Shepherd	M	1.1
D118	Labrador Retriever	F	1.0
D119	Labrador Retriever	M	0.9
D120	Labrador Retriever	F	0.9
D121	Labrador Retriever	F	0.9
D122	Labrador Retriever	M	1.1
D123	Labrador Retriever	M	1.0
D125	Labrador Retriever	F	1.0
D126	Labrador Retriever	F	1.0
